# Common patient‐reported sources of cancer‐related distress in adults with cancer: A systematic review

**DOI:** 10.1002/cam4.7450

**Published:** 2024-07-11

**Authors:** Jennifer M. Stevens, Kathleen Montgomery, Megan Miller, Seyedehtanaz Saeidzadeh, Kristine L. Kwekkeboom

**Affiliations:** ^1^ School of Nursing University of Wisconsin‐Madison Madison Wisconsin USA; ^2^ Edmonton Clinic Health Academy Edmonton Alberta Canada

**Keywords:** cancer‐related distress, concerns, problem list, sources contributing to cancer‐related distress

## Abstract

**Background:**

Cancer‐related distress (CRD) is widely experienced by people with cancer and is associated with poor outcomes. CRD screening is a recommended practice; however, CRD remains under‐treated due to limited resources targeting unique sources (problems) contributing to CRD. Understanding which sources of CRD are most commonly reported will allow allocation of resources including equipping healthcare providers for intervention.

**Methods:**

We conducted a systematic review to describe the frequency of patient‐reported sources of CRD and to identify relationships with CRD severity, demographics, and clinical characteristics. We included empirical studies that screened adults with cancer using the NCCN or similar problem list. Most and least common sources of CRD were identified using weighted proportions computed across studies. Relationships between sources of CRD and CRD severity, demographics, and clinical characteristics were summarized narratively.

**Results:**

Forty‐eight studies were included. The most frequent sources of CRD were worry (55%), fatigue (54%), fears (45%), sadness (44%), pain (41%), and sleep disturbance (40%). Having enough food (0%), substance abuse (3%), childbearing ability (5%), fevers (5%), and spiritual concerns (5%) were infrequently reported. Sources of CRD were related to CRD severity, sex, age, race, marital status, income, education, rurality, treatment type, cancer grade, performance status, and timing of screening.

**Conclusions:**

Sources of CRD were most frequently emotional and physical, and resources should be targeted to these sources. Relationships between sources of CRD and demographic and clinical variables may suggest profiles of patient subgroups that share similar sources of CRD. Further investigation is necessary to direct intervention development and testing.

## Graphical abstract

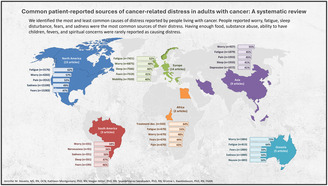

We identified the most and least common causes of distress reported by people living with cancer. People reported worry, fatigue, sleep disturbance, fears, and sadness were the most common sources of their distress. Having enough food, substance abuse, ability to have children, fevers, and spiritual concerns were rarely reported as causing distress. Cancer care teams can use this information to ensure they ask patients about common sources of distress and to develop better ways to support people with cancer if distress becomes a problem.

## 1 BACKGROUND

Nearly everyone with cancer experiences cancer‐related distress (CRD) to some degree. CRD is defined by the National Comprehensive Cancer Network (NCCN) as:

a multifactorial unpleasant experience of a psychological (i.e., cognitive, behavioral, emotional), social, spiritual, and/or physical nature that may interfere with one's ability to cope effectively with cancer, its physical symptoms, and its treatment. Distress extends along a continuum, ranging from common normal feelings of vulnerability, sadness, and fears to problems that can become disabling, such as depression, anxiety, panic, social isolation, and existential and spiritual crisis.^1^

It is estimated that approximately half of all people with cancer experience severe or prolonged CRD,^2^, ^3^ which has been associated with poor outcomes, including greater healthcare utilization, mental health problems, poor quality of life, nonadherence to treatment, disease progression, and higher rates of mortality compared with non‐distressed groups.^4^, ^5^, ^6^, ^7^, ^8^, ^9^

Screening for CRD has been a point of emphasis since its proposal as a standard of care in 1999,^10^ but, to date, has focused nearly exclusively on CRD severity. Identification of ‘actionable’ CRD (CRD requiring intervention) is determined by patient‐reported severity that meets or exceeds a clinical cut‐off, for example, ≥4 on the 0–10 NCCN Distress Thermometer (DT). People with actionable CRD are typically referred to a social worker or health psychologist for further triage, a process which can delay intervention and is declined by >50% of people experiencing CRD.^11^, ^12^, ^13^ Moreover, reluctance to report CRD and accept referrals has been reported if there is a perception that needed resources are limited or unavailable,^12^, ^13^ disclosure could be stigmatizing, or could result in unwanted referral for mental/psychosocial healthcare.^12^ Moving focus from CRD severity to sources of CRD could have utility in identifying risk and improving management of CRD. For the purposes of this paper, “sources of CRD” refers to the specific problems or concerns that contribute to the experience of CRD, such as those identified on the NCCN Distress Thermometer Problem List (PL).

Some organizations attempt to simplify CRD screening by using depression or anxiety measures but fail to capture other sources of CRD.^14^ CRD screening measures should provide people with cancer opportunity to report sources of CRD, allowing healthcare professionals to provide targeted interventions at the time of screening, and/or timely referral for management of specific sources of CRD, (e.g., physical or emotional symptoms, relationship problems, unmet practical needs, existential concerns).

While capturing the source(s) of CRD is critical to providing appropriate intervention, the variety of CRD sources listed on commonly used measures like the PL or the Canadian Problem Checklist (CPC) have been identified as a barrier to CRD screening and management.^15^ Healthcare and community organizations may not have resources needed to offer interventions for the wide range of CRD concerns people may report. Since its introduction, there have been several iterations of the PL, including 46 international adaptations or translations, which have increased the number and variety of sources of CRD from 19 to upwards of 35. International versions of the PL include additions or modifications to items reflecting differences in cultures and healthcare systems, such as access to care, transportation, insurance/financial systems, etc.^16^

Empirical evidence regarding which sources of CRD to include on screening PLs has been limited to a few single site studies or quality improvement projects, which limits generalizability of findings. A study attempting to refine the UK version of the PL added sources to the physical, practical, and spiritual domains based on sources of CRD which were most frequently reported in a sample of 395 patients.^17^ Using a larger sample (*n* = 11,155 medical records), guideline authors^18^ reference a conference presentation citing common concerns in emotional (72.5%), physical (67.5%), and practical domains (43.1%).^19^ Frequency of general domains of CRD is helpful information yet precludes the ability of healthcare providers to be prepared to intervene for specific concerns and increases reliance on referrals for continued triage. Finally, an Australian study aiming to alleviate the burden of CRD assessment used principal component analysis to reduce the number of items on the PL. The authors proposed a two‐item PL consisting of the main components, worry and depression.^15^ Such a PL would likely fail to capture CRD from sources other than anxiety or depression.^14^ With limited aggregate data to rely on, sources of CRD appearing on PLs are largely based upon lower‐level evidence and expert consensus among panel members.^1^, ^20^ To our knowledge, this is the first systematic examination of global evidence regarding frequency of reported sources of CRD.

The purpose of this systematic review was to identify the most (and least) common patient‐reported sources of CRD and associated characteristics in adults with cancer. Our aims were to: (1) describe which patient‐reported sources of CRD are most and least frequently reported in the literature globally and by continent, (2) examine reported relationships between sources of CRD and actionable levels of CRD severity; and (3) examine reported relationships between sources of CRD and clinical and demographic characteristics. Understanding reported sources of CRD can help guide allocation of resources toward interventions for frequently reported sources of CRD and/or those shown to predict actionable levels of CRD severity. Knowledge of associations between sources of CRD and demographic and clinical characteristics can aid in the identification of individuals at risk for actionable levels of CRD and allow clinicians to intervene proactively.

## 2 METHODS

### 2.1 Search strategy

In consultation with a health sciences librarian, the lead author (JS) searched PubMed, PsycINFO, CINAHL, and Scopus databases from January 1995 (3 years before publication of the first NCCN distress management guideline) to February 2023. Search terms included: (distress OR “distress thermometer” OR “distress screener” OR “CPC”) AND (frequen* OR prevalen* OR “commonly reported”) AND psych* AND (oncolog* OR cancer OR neoplasm OR malign* OR tumor). We filtered results by publication type (all academic journals), language (English), and age groups (adult: 19–44 years, middle aged: 45‐64 years, aged: 65+ years, aged, 80 and over).

### 2.2 Inclusion and exclusion criteria

Empirical studies of adults (≥18 years of age), diagnosed with cancer, screened for CRD with the PL or similar measure, and reporting the specified sources of CRD were included. We excluded studies which focused solely on specific subtypes of distress (symptom distress, moral distress, etc.) which differ conceptually from CRD. We also excluded qualitative studies, as they did not provide information about frequency of CRD sources or quantify associations between sources of CRD, actionable CRD, and demographics or clinical characteristics. Articles which collected limited PL data from select domains were also excluded.

### 2.3 Data collection process

The lead author screened titles, abstracts, and full text articles with at least one other member of the research team. Disagreements were discussed among the coauthors until consensus was reached. Data extraction included study characteristics (study design, setting, and location; sample size, cancer diagnosis, stage, treatment type and status), CRD screening information (CRD screening measure, PL version, cut‐off used, proportion of sample reporting actionable CRD, proportion of the sample reporting each source of CRD), and significant reported relationships between sources of CRD and actionable CRD severity, demographics, and clinical variables. Baseline data was extracted from longitudinal studies. All data were extracted by the lead author into a spreadsheet created for this review. Data were reviewed and verified for accuracy by at least one other study team member. Disagreements were resolved through additional review and discussion with coauthors until consensus was reached.

### 2.4 Data analysis

#### 2.4.1 Aim 1

We computed a weighted proportion for each source of CRD to evaluate its relative frequency across studies. For each CRD source, we computed the number of persons endorsing the concern (multiplying the percentage reported by the study's sample size, if necessary), then summed the number endorsing across studies and divided by the pooled sample size. We analyzed findings globally, then repeated the analyses at the continent level. Sources of CRD were organized into (1) standard sources of CRD according to the NCCN PL, version 2.2021, and (2) additional sources of CRD found on modified PLs and other measures.

#### 2.4.2 Aims 2 and 3

Statistically significant relationships between CRD domains or individual sources of CRD and actionable levels of CRD severity, demographics, and clinical characteristics were analyzed narratively. We organized relationships by CRD domain and summarized findings with respect to the direction of the relationship.

### 2.5 Quality appraisal

The Mixed Methods Appraisal Tool (MMAT) was used to assess quality of included studies.^21^ We rated each study on five criteria based on type of study design. Studies meeting at least four of the five criteria, and achieving a rating of ≥80%, were considered high quality. Ratings of 40%–60% were considered moderate, and ratings below 40% were considered poor quality. We did not exclude articles based on quality reporting for aim 1, which focused on descriptive data. Findings from studies of low quality were excluded for aims 2 and 3, as risk of bias jeopardizes validity of the relationships that were subject to statistical tests.

## 3 RESULTS

### 3.1 Study selection

**FIGURE 1:**
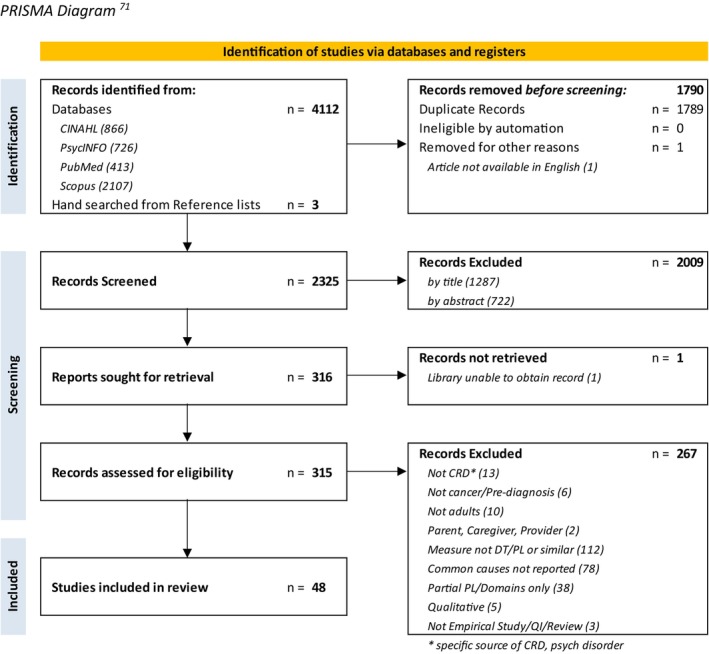
PRISMA diagram.^22^

A total of 2324 articles were screened for eligibility, 2321 yielded from search results after removing duplicates and three articles identified through hand searching reference lists. Forty‐eight articles of unique studies met inclusion criteria and were included in this review (see PRISMA diagram, Figure 1). A table summarizing data from all included studies is presented in Table 1.

**TABLE 1:** Literature review table, organized by cancer type.

Authors	*n*	Measure	Most common domain	Most common sources (top three)	Least common sources (last three)	Associations between actionable CRD and sources of CRD	Sources of CRD associated with demographic characteristics	Sources of CRD associated with clinical characteristics
*Multiple cancer types*								
Abd El‐Aziz et al., 2020^23^	550	DT/PL Arabic 36‐item PL	NR	Treatment decisions (64%) Worry (47%) Fears (45%)	Spiritual (4%) Work and School (6%) Substance Abuse (6%)	Concerns significantly associated with actionable CRD were: Health insurance (OR 7.6) Depression (OR 6.9) Worry (OR 4.4) Fear (OR 3.9) Sleep disturbance (OR 3.6) Loss of interest (OR 3.1) Sadness (OR 2.0)	NR	NR
Ascendio‐Huertas et al., 2021^24^	646	DT /PL 40‐item PL v. 1.2008	NR	Feeling swollen (71%) Fatigue (68%) Worry (66%)	Spiritual / Religious (7%) Sexual (7%) Dealing with Partner (8%)	Concerns significantly associated with actionable CRD were: Housing (OR 2.6) Sadness (OR 2.5) Eating (OR 1.7) Transportation (OR 1.7) Nervousness (OR 1.5)	NR	NR
Bellè et al., 2016^25^	102	DT/PL Italian 36‐item PL	Physical	Worry (75%) Fears (49%) Nervousness (40%)	Mouth Sores (2%) Housing (4%) Breathing (4%)	Greater number of endorsements was associated with actionable CRD in emotional (*p* < 0.001), family (*p* = 0.014), physical (*p* = 0.039), and spiritual (*p* = 0.046) domains Endorsement of practical concerns was not associated with actionable CRD	Number of reported sources in any domain of CRD did not significantly differ by sex	Number of reported sources in any domain of CRD did not differ significantly by diagnosis
Bergerot et al., 2018^26^	137	DT/PL Portuguese 35‐item PL v.1.0	Emotional and Physical	Worry (59%) Pain (51%) Nervousness (45%)	Eating (25%) Transportation (26%) Breathing (26%)	Actionable CRD was related to reporting physical (93%) and emotional (74%) sources of CRD	NR	NR
Blais et al., 2014^27^	911	DT/CPC 21 items	Emotional	Fears (51%) Coping (44%) Sleep disturbance (42%)	Relating to God (7%) Loss of Faith (8%) Housing (8%)	Greater number of endorsements is associated with actionable CRD in all CPC domains (*p* < 0.001)	Women reported more concerns than men overall (*p* < 0.001), and in specific domains of emotional (*p* < 0.001), social (*p* < 0.001), spiritual (*p* < 0.001), and informational (*p* = 0.008)	NR
Ebob et al., 2022^28^	120	DT/PL 35‐item PL v. 3.2019		Insurance/ Financial (73%) Fatigue (69%) Transportation (61%)	NR	NR	NR	NR
Giese‐Davis et al., 2012^29^	1196^a^	DT/PCL 20 items	Psycho‐social^b^	Worry (42%) Sleep disturbance (34%) Being a burden to others (29%)	NR	Endorsement of psychosocial concerns was associated with actionable CRD at baseline (*r* = 0.386, *p* < 0.001) and 12 months (*r* = 0.266, *p* < 0.001)	Age (younger) and sex (female) predicted greater number of psychosocial endorsements (*p* < 0.001; *p* = 0.02) Single people reported more practical concerns than married people Females reported more psychosocial concerns than males Changes in number of psychosocial concerns reported concerns over time: Number of endorsements decreased over time (*p* < 0.0001) Married patients improved more (reported fewer concerns over time) than single patients (*p* = 0.04) Older female patients and younger male patients reporting psychosocial sources improved more over time than younger females and older males (three‐way interaction; *p* = 0.04)	NR
Guan et al., 2019^30^	441	DT/PL Chinese 40‐item PL	Physical	Pain (71%) Constipation (43%) Sleep disturbance (37%)	NR	People reporting emotional concerns had significantly higher odds of reporting actionable CRD (OR = 2.667, *p* < 0.001). No other domains were significant. People reporting pain (breakthrough or severe pain) had significantly higher odds of reporting actionable CRD (OR = 2.402, *p* < 0.001; OR 1.163, *p* = 0.004)	NR	NR
Hildenbrand et al., 2022^31^	848	DT/PL 35‐item PL v. 3.2019		Pain (79%) Fatigue (87%) Eating (73%)	NR	Authors observed people with actionable CRD reported proportionally more concerns on the PL than those with non‐actionable CRD.	NR	NR
Hollingworth et al., 2013^32^	112^a^ DT/PL group	DT/PL 38‐item PL v. 2012	Physical	Fatigue (61%) Fears (39%) Sleep disturbance (35%)	NR	NR	NR	NR
Hong et al., 2015^33^	153	DT/PL Chinese 36‐item PL	Emotional	Worry (74%) Depression (56%) Pain (54%)	Fevers (3%) Spiritual (3%) Work/ School (6%)	NR	NR	NR
Kirk et al., 2021^34^	1071	DT/PL 39‐item v. 2.2020	Emotional	Worry (82%) Fear (57%) Sadness (56%)	Family health issues (15%) Appearance (16%) Dealing with children (21%)	Emotional concerns were most associated with actionable CRD (four of five significant concerns were in the emotional domain; 95% CI) Concerns significantly associated with actionable CRD (95% CI) include: Worry (OR 3.6) Depression (OR 1.8) Sadness (OR 1.7) Lack of control over treatment decisions (OR 1.55) Loss of interest (OR 1.36)	NR	Referral to information services was associated with reporting worry (OR 2.07) or nervousness (OR 1.83) Referral to emotional health services was associated with reporting depression (OR 1.75) Referral to practical services was associated with reporting financial/insurance (OR 15.57) or memory/concentration (OR 1.81)
Kyranou et al., 2020^35^	152	DT/PL Greek 40‐item PL	Physical	Fatigue (55%) Worry (49% Numbness or tingling in hands/feet (38%)	Substance abuse (2%) Ability to have children 6% Fevers (8%)	Greater number of endorsements is associated with actionable CRD Concerns associated with actionable CRD (*p* ≤ 0.01): treatment decisions, family health issues, depression, fears, nervousness, sadness, worry, loss of interest, breathing, diarrhea, eating, fatigue, getting around, nausea, pain, sleep disturbance Concerns associated with actionable CRD (*p* ≤ 0.05): housing, insurance/financial, dealing with partner, appearance, bathing/ dressing, constipation, feeling swollen, fevers, nose dry/congested, skin dry/itchy	NR	NR
Mahendran et al., 2017^36^	44	DT/PL 34‐item PL No version specified	Emotional	Worry (46%) Nervousness (26%) Sadness (24%)	Dealing with Partner (2%) Relating to God (4%) Others NR	Greater number of endorsements is associated with actionable CRD Emotional concerns were most associated with severe distress Nervousness (*p* = 0.02) Sadness (*p* = 0.05) Changes in Urination (*p* = 0.03) Constipation (*p* = 0.03)	Fear (*p* = 0.045) and sadness (*p* = 0.039) were more frequently reported among people aged 41–50. Female sex was associated with higher number of reported sources of CRD Females reported more family concerns than males People with non‐formal education reported more emotional concerns—fears (*p* = 0.046) and nervousness (*p* = 0.039) More physical concerns were reported by Malays than other ethnic groups (*p* = 0.013)	People reported more physical concerns before seeing the oncologist than after consultation (*p* = 0.003) More emotional concerns reported if pt had not had surgery or radiation therapy before the first chemo treatment (*p* = 0.018) People who did not have surgery reported more physical concerns (*p* = 0.008) and worry (*p* = 0.027) than people who had surgery
Mendonça et al., 2021^37^	91	Dt/PL 35‐item PL v. 2013		Worry (75%) Nervousness (56%) Insurance/ Financial (52%)	Fevers (5%) Mouth Sores (8%) Dealing with children (9%)	NR	NR	NR
McDonagh et al., 2018^38^	82	DT/PL version not specified	NR	Fears (44%) Fatigue (40%) Pain (38%)	Legal Issues (2%) Ability to have Children (4%) Substance Use (5%)	Emotional concerns were most associated with severe distress	African Americans reported significantly greater numbers of practical (*p* = 0.004) and spiritual (*p* = 0.006) concerns than Caucasians Specific concerns reported more frequently by African Americans include: Insurance (*p* < 0.001) Transportation (*p* < 0.001) Work/School (p = 0.044) Changes in urination (*p* = 0.005) Memory/concentration (*p* = 0.004) Fertility (*p* = 0.01) Sexual (*p* = 0.044)	NR
Mehnert et al., 2018^39^	3724	DT/PL German 34‐item PL	Physical	Fatigue (56%) Sleep disturbance (51%) Worry (47%)	Childcare (2%) Loss of Faith (3%) Spiritual (5%)	Greater number of endorsements is associated with actionable CRD (*p* < 0.001) Specific concerns associated with higher mean CRD severity include sadness, fatigue, sleep disturbance, and getting around. People reporting sadness, fatigue, and sleep disturbance together had higher mean CRD severity than those reporting only one of those three sources of CRD	Females reported most physical and psychosocial concerns more frequently than males (*p* < 0.05) Males reported sexual concerns and changes in urination more frequently than females (*p* < 0.05) Females reporting worry had higher mean CRD severity than males reporting worry	Number of reported sources of CRD did not differ by treatment setting
Musiello et al., 2017^40^	68	DT/PL 40‐item PL v.1999	Physical	Fatigue (65%) Worry (49%) Fears (40%)	Spiritual (2%) Others NR	Greater number of endorsements is associated with actionable CRD	NR	NR
Nguyen et al., 2021^41^	300	DT/PL Modified Vietnamese 42‐item PL Added cognitive & information sources	NR	Memory/ Conc. (59%) Sleep disturbance (59%) Nervousness (51%)	Facing mortality (2%) Meaning/ purpose in life (2%) Dealing with partner (2%)	Specific concerns associated with actionable CRD include: Housing (OR = 0.4) Insurance (OR = 0.4) Work/school (OR = 0.4) Dealing with children or partner (OR = 0.04) Depression (OR = 0.1) Nervousness (OR = 0.2) Boredom (OR = 0.1) Isolation (OR = 0.1) Adjusting to illness (OR = 0.3) Pain (OR = 2.9) Fatigue (OR = 3.9) Insomnia (OR = 2.4) Breathing (OR = 3.4) Loss of appetite (OR = 1.6)	NR	NR
Opie et al., 2017^42^	242	DT/PL 37‐item PL v.1.2016	NR	Fatigue (59%) Worry (45%) Sleep disturbance (42%)	NR	Greater number of endorsements is associated with actionable CRD Sources most significantly associated with actionable CRD (*p* < 0.001) include: Practical: insurance, work, housing, and dealing with partner Emotional: fears, sadness, loss of interest, depression, and worry Physical: fatigue, pain, and appearance Other sources significantly associated with actionable CRD: Transportation (*p* < 0.04) Dealing with children (*p* < 0.01) Sleep disturbance (*p* < 0.01) Memory/ concentration (*p* < 0.002) Constipation (*p* < 0.004) Nausea (*p* < 0.05)	NR	NR
Peters et al., 2020^43^	1735	DT/PL German 34‐item PL	NR	Fatigue (47%) Fears (47%) Worry (46)	Spiritual (3%) Dealing with children (5%) Dealing with partner (6%)	Endorsement of any one source of fatigue, fears, or worry (OR = 3.51) If all three (fatigue, fears, and worry) were reported, OR = 23.9	Women reported more emotional (*p* < 0.001), physical (*p* < 0.001), and (*p* = 0.003) practical concerns than men People under 50 reported more practical concerns than people aged 51–64; people over 65 reported fewer practical concerns than people aged 51–64 People under 65 reported more family and emotional concerns than people over 65 years of age	NR
Roerink et al., 2013^16^	145	DT/PL Dutch 47‐item PL	Physical	Physical Fitness (47%) Sleep disturbance (42%) Muscle Strength, Nervousness (40%)	Fevers (1%) Housing (6%) Changes in Urination, Diarrhea (8%)	Greater number of overall endorsements is associated with actionable CRD (*p* < 0.001) Greater number of endorsements in each domain was significantly correlated with actionable CRD: emotional (*p* < 0.001), practical (*p* < 0.001), family (*p* < 0.001), physical (*p* < 0.001), and spiritual (*p* = 0.006)	NR	NR
Saedi et al., 2012^44^	265	DT/PL modified 34‐item PL Physical Concerns divided into three groups	Physical	Sleep disturbance (51%) Depression (50%) Fatigue (48%)	Housing, Relating to God (4%) Bathing (5%) Fevers (6%)	Concerns most significantly associated with actionable CRD (*p* ≤ 0.001) include: Practical: childcare Family: dealing with children, dealing with partner Emotional: depression, fears, nervousness Physical: appearance, eating, fatigue, getting around, indigestion, nausea, numbness/tingling, pain, skin dry/itchy, sleep disturbance Other concerns significantly associated with actionable CRD: Bathing/dressing (*p* = 0.002) Concentration (*p* = 0.03) Diarrhea (*p* = 0.02) Fever (*p* = 0.01) Nose congested/dry (*p* = 0.004) Urination (*p* = 0.02)	NR	NR
Tonsing et al., 2018^45^	43	DT/PL 39‐item PL v.2009	Physical	Pain (33%) Fatigue (30%) Nervousness, Transportation, Treatment Decisions (28%)	Housing (7%) Dealing with partner, Getting around, Loss of Interest, Spiritual (9%) Congested, Depression (12%)	People with actionable CRD reported more concerns in emotional and physical domains (*p* < 0.01) Specific sources of CRD associated with actionable CRD included: Pain (*p* ≤ 0.01) Sleep disturbance (*p* ≤ 0.01) Fears (*p* ≤ 0.05) Nervousness (*p* ≤ 0.05) Sadness (*p* ≤ 0.05) Worry (*p* ≤ 0.05) Fatigue (*p* ≤ 0.05)	NR	NR
VanHoose et al., 2015^46^, ^c^	1205	DT/PL 36‐item PL v. 1.2008	Physical	Worry (68%) Financial / Insurance (60%) Fears, Nervousness, Sleep disturbance (48%)	Loss of faith (2%) Childcare (5%) Facility (parking & wait time; 7%)	Concerns significantly associated with actionable CRD: Worry (OR = 5.6) Getting Around (OR = 3.5) Financial (OR = 2.5) Sleep disturbance (OR = 1.9) Nervousness (OR = 1.8)	NR	NR
Watts et al., 2016^47^	441	DT/PL 39‐item PL v. 2013	Physical	Fatigue (70%) Worry (67%) Sadness (53%)	Substance use (1%) Ability to have children (2%) Spiritual (4%)	NR	Differences in remoteness categories was found for the following sources of CRD: Insurance/financial concerns (p = 0.04) Transportation (*p* = 0.004) Fears (*p* = 0.001) Sadness (*p* = 0.002) Worry (*p* < 0.001) Mouth sores (*p* = 0.03) Nausea (*p* = 0.04) Pain (*p* = 0.02)	NR
Wookey and McKean, 2016^48^	40	DT/PL 39‐item PL v. 2014	Physical	Nervousness (71%) Worry (58%) Fears (48%)	Ability to have children, changes in urination, dealing with partner, housing, spiritual (3%) Sexual (5%) Breathing, childcare, diarrhea, nausea, swelling, (8%)	Concerns significantly associated with actionable CRD: Depressed mood (*p* < 0.001) Fears (*p* < 0.001) Sleep disturbance (*p* = 0.001) Worry (*p* = 0.003) Fatigue (*p* = 0.006) Nervousness (*p* = 0.015) Eating (*p* = 0.024) Loss of interest (*p* = 0.029)	NR	NR
Zainal et al., 2007^49^	168	DT/PL Malaysian 40‐item PL v.1999	Physical	Fatigue (48%) Worry (47%) Sleep disturbance (39%)	Diarrhea (1%) Bathing (6%) Fevers & Spiritual (7%)	NR	NR	NR
*Brain/CNS cancers*								
Goebel et al., 2011^50^	159	DT/PL German 40‐item PL	Physical	Fatigue (54%) Fears (48%) Worry (46%)	Loss of Faith (1%) Childcare (3%) Breathing, Fevers, Mouth Sores, and Sexual Concerns (4%)	Greater number of endorsements is associated with actionable CRD Greater number of endorsements in the following domains was significantly correlated with actionable CRD: emotional (*p* < 0.001), practical (*p* < 0.001), physical (*p* < 0.001), and spiritual (*p* = 0.026)	No relationship between sex and number of concerns reported Younger age associated with more endorsements on PL	Performance status was negatively correlated with the number of practical concerns reported No significant associations between the number of concerns reported and cancer stage, time since diagnosis, or type of surgery
Randazzo et al., 2017^51^	829	DT/PL Dutch 47‐item PL	Physical	Fatigue (43%) Memory/Concentration (41%) Worry (29%)	Fevers (1%) Spiritual, Ability to have children (2%) Breathing (4%)	NR	Being female was associated with higher number of endorsements on PL (*p* = 0.004), specifically in practical (*p* = 0.003), family (*p* = 0.021), emotional (*p* < 0.001), and physical (*p* = 0.023) domains. The only concern reported by greater proportion of men than women was sexual concerns	Significantly higher percentage of problems were reported <1 year from diagnosis compared with than >1 year from diagnosis in practical (*p* = 0.002), emotional (*p* < 0.001), and physical (*p* < 0.001) domains WHO Grade of “undetermined” was associated with higher proportion of physical concerns (*p* = 0.047)
Rooney et al., 2013^52^	154^a^	DT/PL 37‐item PL v. 2012	Physical	Fatigue (35%) Sleep disturbance (31%) Worry (29%)	Fevers, Relating to God, Loss of Faith (2%) Childcare (4%) Dealing with Children, Sexual (5%)	Greater number of endorsements is associated with actionable CRD (*p* < 0.001)	Males reported more concerns than females (*p* = 0.036)	NR
*Breast cancer*								
Budisavljevic et al., 2021^53^	201	DT/PL 41‐item PL v 2.2020	NR	Worry (58%) Fatigue (35%) Sleep disturbance (33%)	Fevers (1%) Bathing (2%) Sexual (3%)	Endorsement of emotional concerns were most associated with actionable CRD Emotional concerns: Worry (*p* < 0.001) Nervousness (*p* < 0.001) Fear (*p* < 0.001) Loss of interest (*p* = 0.001) Sadness (*p* = 0.003) Depression (*p* = 0.004) Practical Concerns: Work/school (*p* = 0.001) Housing (*p* = 0.01) Childcare (*p* = 0.017) Physical Concerns: Pain (*p* < 0.001) Fatigue (*p* < 0.001) Sleep disturbance (*p* < 0.001) Memory/Concentration (*p* < 0.001) Tingling in hands/feet (*p* = 0.001) Eating (*p* = 0.002) Skin dry/itchy (*p* = 0.005) Appearance (*p* = 0.006) Diarrhea (*p* = 0.022) Indigestion (*p* = 0.022) Mouth sores (*p* = 0.036) Feeling swollen (*p* = 0.042) No family or spiritual concerns were significantly related to actionable CRD	Concerns about childcare were associated with education level (*p* < 0.05)	Concerns about childcare were associated with receiving treatment other than chemotherapy (*p* < 0.05)
Hegel et al., 2006^54^	236	DT/PL modified 34‐item PL v. 2001 added Uncertainty	Emotional	Uncertainty (96%) Worry (89%) Fears (82%)	NR	NR	NR	NR
Mertz et al., 2012^55^	357	DT/PL Danish 35‐item PL	NR	Nervousness (71%) Sleep disturbance (50%) Memory/Concentration (42%)	Fevers (1%) Mouth Sores, Spiritual (2%) Bathing (3%)	Greater number of endorsements is associated with actionable CRD, adjusted for age (OR 1.83, DT ≥3; OR 1.37, DT ≥7) People with actionable CRD reported significantly more concerns in emotional, practical, and physical domains (*p* < 0.05)	NR	NR
Naik et al., 2020^56^	10,734	CPC 21 items	NR	Sadness (52%) Fears/Worries (49%) Understand Illness (39%)	Intimacy/Sexuality (6%) Faith (6%) Meaning/Purpose in life (6%)	NR	Young adults (age <39) were found to be significantly more likely to report specific sources of CRD than those aged over 39 (*p* < 0.001): Work/School (OR = 3.79) Intimacy/Sexuality (OR = 2.82) Finance (OR = 2.78) Fear/Worry (OR = 2.63) Frustration/Anger (OR = 2.28) Change in appearance (OR = 2.25) Sadness (OR = 2.18) Worry about family (OR = 2.08) Feeling a burden to others (OR = 1.88) Making treatment decisions (OR = 1.52) Knowing resources (OR = 1.51)	NR
*GI Cancers*								
Hong et al., 2015^57^	165	DT/PL Chinese 36‐item PL	Physical	Pain (*n* = 103) Worry (*n* = 101) Indigestion (*n* = 98) unable to calculate %s	NR	Actionable CRD was significantly (p ≤ 0.001) associated with endorsement of stomach pain (*r* = 0.273), eating restrictions (*r* = 0.253), and anxiety (*r* = 0.584).	NR	NR
Jacobs et al., 2017^58^	187	DT/PL Dutch 47‐item PL	NR	Eating (75%) Tension (61%) Weight Change (58%)	NR	Greater number of endorsements is associated with actionable CRD Total number of Physical concerns (OR 1.5) Total number of emotional concerns (OR 1.2) Total number of practical concerns (OR 0.4) Fatigue (OR 0.63) Pain (OR 3.37)	Being female was associated with higher number of endorsements on PL (OR 1.63)	NR
*Gynecologic cancers*								
Jewett et al., 2020^59^	355	DT/PL 40‐item PL v. 1.2016	Physical	Fatigue (54%) Worry (50%) Sleep disturbance (46%)	Substance Abuse (1%) Fevers (2%) Ability to have children (3%)	Greater number of endorsements is associated with actionable CRD.	Odds of reporting fatigue, sadness, nervousness, fears, and sadness differed by: Older age decreased odds (per 5 years; 0.77–0.86) Lower household income increased odds <$50,000 (OR 3.01–3.86); $51,000–$100,000 (OR 1.28–2.48) Having a partner increased odds of reporting nervousness (OR 1.95)	Receiving chemotherapy increased odds of reporting: pain (OR 3.66), fatigue (OR 2.51), sadness (OR 2.39), fears (OR 2.01), and nervousness (OR 1.67). Cervical cancer diagnosis decreased odds of reporting pain as a source of CRD compared with uterine cancer (OR 0.19).
O'Connor et al., 2017^60^, ^c^	62	DT/PL Modified v. 2003, added cognitive items	Physical	Nervousness (63%) Worry (53%) Fears (50%)	NR	Greater number of endorsements is associated with actionable CRD (*r* = 0.53, *p* = 0.0005)	NR	NR
Seland et al., 2022^61^	92	DT/PL Norwegian v. 2.2017	NR	Fatigue (58%) Tingling in hands/feet (54%) Worry (53%)	Fevers (1%) Spiritual (2%) Substance Abuse (2%)	Greater number of endorsements is associated with actionable CRD (*p* < 0.001) Concerns significantly associated with actionable CRD: Dealing with partner (*p* = 0.02) Sadness (*p* < 0.001) Worry (*p* < 0.001) Depression (*p* = 0.02) Fears (*p* < 0.001) Nervousness (*p* = 0.002) Loss of interest (*p* = 0.007) Appearance (*p* = 0.02) Memory/concentration (*p* = 0.006) Pain (*p* = 0.02) Sexual function (*p* = 0.02) Sleep disturbance (*p* = 0.03) Physical endurance (*p* < 0.001) Muscle strength (*p* < 0.001)	NR	NR
*Hematologic cancer*								
Bergerot et al., 2015^62^	104^a^	DT/PL 35‐item PL v. 2013	Physical	Worry (74%) Sadness (69%) Sleep disturbance (62%)	Childcare (1%) Transportation (7%) Housing; Spiritual (11%)	Greater number of endorsements is associated with actionable CRD Actionable CRD related to emotional (*p* < 0.001), practical (*p* < 0.0001), family (*p* = 0.003), and physical (*p* < 0.0001) concerns decreased over time Decrease in actionable CRD over time differed between men and women related to physical (*p* = 0.046) and family (*p* = 0.048) concerns	Males reported more practical, concerns than women at baseline, but women reported more practical concerns than men at mid and end study points Women reported more concerns, particularly emotional and physical concerns than men Patients reported fewer physical concerns over time	People with leukemia reported more practical concerns initially, but people with lymphoma reported more practical, family, and spiritual concerns at mid and end study points People with myeloma reported more family and spiritual concerns at baseline People with high grade cancer reported more practical concerns No significant associations between sources of CRD and stage of disease
Braamse et al., 2014^63^	247	DT/PL Dutch 47‐item PL	Physical	Physical Fitness (61%) Fatigue (59%) Tingling in hands/feet (42%)	NR	NR	Younger age associated with more endorsements on PL (*p* = 0.01) Female sex was associated with higher number of endorsements on PL (*p* = 0.05)	Number of decreased significantly (*p* < 0.001) over time after allogenic stem cell transplant: 0–1 year (15 concerns), 1–2.5 years (13 concerns), and 2.5–5.5 years (8 concerns) Experience of graft versus host condition in allogenic stem cell transplant recipients was associated with reporting significantly more concerns (*p* = 0.03) No difference between number concerns reported and treatment type
Park et al., 2022^64^	132	DT/PL 39‐item PL v. 2.2020	NR	Fatigue (61%) Worry (60%) Numbness or tingling in hands/feet (60%)	Work/school (1%) Childcare (2%) Sexual (3%)	Concerns significantly associated with actionable CRD in univariate analysis: Transportation (*p* = 0.003) Treatment decisions (*p* = 0.04) Housing (*p* = 0.018) Dealing with partner (*p* = 0.02) Worry (*p* < 0.001) Fear (*p* < 0.001) Sadness (*p* = 0.02) Nervousness(*p* < 0.001) Loss of interest (*p* < 0.001) Pain (*p* = 0.005) Constipation (*p* = 0.01) Eating (*p* = 0.005) Transportation (*p* = 0.042), depression (*p* = 0.001), and constipation (*p* = 0.021) remained significantly associated with actionable CRD in multivariate analysis.	NR	NR
Troy et al., 2019^65^	304	DT/PL 39‐item PL v.2017	Physical	Fatigue (614 end) Pain (417 end) Worry (384 end)	NR	NR	NR	NR
*Lung cancer*								
Graves et al., 2007^66^	333	DT/PL Modified 42‐item PL Added cognitive & information items	Physical	Fatigue (56%) Pain (51%) Nervousness (49%)	NR	The most common symptoms reported among people with actionable CRD (DT ≥4) were: Fatigue (65.4%) Pain (64.9%) Nervousness/anxiety (62.4%) Breathing (52.7%) Depression/sadness (49.3%) Sleep disturbance/insomnia (48.3%) Concerns shown to be significant predictors of actionable CRD (multiple R^2^ 29.5%) included: Depression/sadness (*β* = 0.232, *p* = 0.036) Nervousness/anxiety (*β* = 0.171, *p* = 0.002) Pain (*β* = 0.189, *p* < 0.001) Fatigue (*β* = 0.141, *p* = 0.007)	NR	Endorsement of greater numbers of concerns was associated with increased desire for help (*p* < 0.001)
Tan et al., 2019^67^	420^a^	DT/PL Chinese 40‐item PL	Physical	Insurance/financial (62%) Worry (50%) Fatigue (47%)	NR	NR	Having lower education (*p* = 0.006) or income (*p* = 0.047) was associated with reporting worry on the PL	Having national insurance compared with urban options (*p* = 0.002), and currently receiving cancer treatment (*p* = 0.0051) was associated with reporting worry on the PL
*Prostate cancer*								
Mehnert et al., 2007^68^	197	DT/PL German 34‐item PL	Physical	Changes in urination, sexual (56%) Getting around, pain (41%) Fatigue (36%)	Childcare (1%) Loss of faith (2%) Nausea, mouth sores, eating (3%)	Greater number of endorsements is associated with actionable CRD (*p* = 0.036) Total number of physical concerns (*p* = 0.001) Fatigue (*p* = 0.001) Sleep disturbance (*p* = 0.001) Getting around (*p* = 0.005) Sexual (*p* = 0.006) Pain (*p* = 0.01) Urination (*p* = 0.02) Fevers (*p* = 0.04) Total number of emotional concerns (*p* = 0.001) Worry (*p* = 0.001) Fears (*p* = 0.001) Nervousness (*p* = 0.001) Sadness (*p* = 0.007) Total number of practical concerns (*p* = 0.004) Work/school (*p* = 0.004)	NR	NR
*Myeloproliferative neoplasms*								
Troy et al., 2018^69^	124^a^	DT/PL 39‐item PL v. 1.2018	Physical	Fatigue (*n* = 181) Pain (*n* = 95) Worry (*n* = 80)	Least reported source of CRD was Spiritual (1 end) No others reported	Greater number of endorsements is associated with actionable CRD (*r* = 0.70, *p* < 0.001)	NR	NR

Abbreviations: CPC, Canadian Problem Checklist; DT, NCCN Distress Thermometer; end, number of endorsement(s); MVF, factor of increase or decrease from multivariate analysis; *n*, number of participants screened; sNR, not reported; PCL, the modified Problem Checklist; PL, NCCN Problem List.

^a^ Baseline n/data used for longitudinal studies.

^b^ PCL psychosocial domain has 13 concerns: burden to others, worry about family/friends, talking with family, talking with medical team, family conflict, changes in appearance; alcohol/drugs, smoking, coping, sexuality, spirituality, treatment decisions and sleep disturbance.

^c^ Data not included in analysis for aim 2 and aim 3.

### 3.2 Study characteristics

Most included studies were quantitative descriptive studies (*n* = 46), with one randomized controlled trial and one mixed methods study. Most were cross‐sectional (*n* = 42). Forty‐four studies were conducted among people with solid tumor cancers, and four studies included people with hematologic malignancies only. More than half of the studies included participants representing multiple cancer types (*n* = 29). Across studies, participants were mainly female (55%), married (72%), and White (79%). Studies were mainly conducted outside of the United States (*n* = 37) in outpatient clinic settings (*n* = 34). The NCCN DT/ PL was the most common measure used (*n* = 40), with eight studies using modified versions or alternate measures (e.g., CPC). Study characteristics are presented in Table 2.

**TABLE 2:** Characteristics of included studies (*n* = 48).

	*n* Studies (%)	M (SD or range)
*Study characteristics*		
Study design		
Quantitative Descriptive Studies	46 (96%)	
Randomized controlled trials	1 (2%)	
Mixed methods	1 (2%)	
Temporality		
Cross‐Sectional	42 (88%)	
Longitudinal	6 (13%)	
Study sample size		634 (40–10,734)
Study origin		
Africa	2 (4%)	
Asia	9 (19%)	
Europe	14 (29%)	
North America	15 (31%)	
Oceania	5 (10%)	
South America	3 (6%)	
Setting		
Outpatient (OP)	34 (71%)	
Inpatient (IP)	6 (13%)	
Other (Public Health, Both OP and IP)	8 (16%)	
*Cancer‐related distress across studies*		
CRD measure used		
NCCN PL	40 (83%)	
Modified NCCN PL	4 (8%)	
CPC	2 (4%)	
Other	2 (4%)	
Cut‐off for Actionable CRD (DT 0–10)	42 (88%)	4 (3–6)
Actionable CRD	38 (91%)	53% (23%–91%)
*Study sample characteristics*		
Age		
Sex—female	48 (59%)	58 (18–93)
Race—White	11 (80%)	
Married	26 (76%)	
Employed	10 (40%)	
Cancer type		
Multiple cancer types	29 (60%)	
Brain	3 (6%)	
Breast	4 (9%)	
Gastrointestinal	2 (4%)	
Gynecologic	3 (6%)	
Lung	2 (4%)	
Hematologic	4 (9%)	
Prostate	1 (2%)	
Stage	NR 15 (31%)	
All (0–IV)	29 (60%)	
Stage I–III	1 (2%)	
Advanced	2 (4%)	
Treatment	NR 17 (31%)	
On treatment	16 (36%)	
Both on and off treatment	13 (29%)	
Before treatment	2 (4%)	

### 3.3 Quality appraisal

The majority of included studies were high quality (*n* = 33). Thirteen studies were rated moderate quality, and two studies were rated low quality.^46^, ^60^ Quality ratings for each of the studies are reported in Table 3.

**TABLE 3:** MMAT quality appraisal (*n* = 48).

	Rating	Sampling strategy is appropriate	Sample is representative	Measures are appropriate	Risk of non‐response bias is low	Statistical analysis is appropriate
Quantitative descriptive studies (*n* = 46)						
Abd El‐Aziz et al.^23^	100%	▲	▲	▲	▲	▲
Ascencio‐Huertas et al.^24^	100%	▲	▲	▲	▲	▲
Bellè et al.^25^	60%	▲	═	▲	═	▲
Bergerot et al.^26^	60%	▲	═	▲	═	▲
Bergerot et al.^62^	100%	▲	▲	▲	▲	▲
Blais et al.^27^	100%	▲	▲	▲	▲	▲
Braamse et al.^63^	80%	▲	▲	▲	═	▲
Budisavljevic et al.^53^	80%	▲	▲	▲	═	▲
Ebob‐Anya et al.^28^	100%	▲	▲	▲	▲	▲
Giese‐Davis et al.^29^	80%	▲	▲	▲	═	▲
Goebel et al.^50^	60%	▲	═	▲	═	▲
Graves et al.^66^	100%	▲	▲	▲	▲	▲
Guan et al.^30^	80%	▲	▲	▲	═	▲
Hegel et al.^54^	80%	▲	▲	▲	Х	▲
Hildenbrand et al.^31^	100%	▲	▲	▲	▲	▲
Hong et al.^33^	60%	▲	═	▲	═	▲
Hong et al.^57^	40%	▲	Х	▲	═	═
Jacobs et al.^58^	100%	▲	▲	▲	▲	▲
Jewett et al.^59^	80%	▲	▲	▲	═	▲
Kirk et al.^34^	80%	▲	▲	▲	Х	▲
Kyranou et al.^35^	80%	▲	▲	▲	═	▲
Mendonça et al.^37^	100%	▲	▲	▲	▲	▲
Mahendran et al.^36^	80%	▲	▲	▲	Х	▲
McDonagh et al.^38^	80%	▲	▲	▲	═	▲
Mehnert et al.^39^	80%	▲	▲	▲	═	▲
Mehnert et al.^68^	80%	▲	▲	▲	═	▲
Mertz et al.^55^	80%	▲	▲	▲	═	▲
Musiello et al.^40^	60%	▲	═	▲	═	▲
Naik et al.^56^	80%	▲	▲	▲	═	▲
Nguyen et al.^41^	80%	▲	▲	▲	═	▲
Opie et al.^42^	40%	═	═	▲	Х	▲
Park et al.^64^	100%	▲	▲	▲	▲	▲
Peters et al.^43^	80%	▲	▲	▲	═	▲
Randazzo et al.^51^	80%	▲	▲	▲	═	▲
Roerink et al.^16^	100%	▲	▲	▲	▲	▲
Rooney et al.^52^	80%	▲	▲	▲	═	▲
Saedi et al.^44^	40%	═	Х	▲	═	▲
Seland et al.^61^	100%	▲	▲	▲	▲	▲
Tan et al.^67^	100%	▲	▲	▲	▲	▲
Tonsing and Vungkhanching^45^	40%	═	═	▲	═	▲
Troy et al.^69^	80%	▲	▲	▲	═	▲
Troy et al.^65^	100%	▲	▲	▲	▲	▲
VanHoose et al.^46^	20%	═	═	▲	═	═
Watts et al.^47^	60%	▲	═	▲	═	▲
Wookey and McKean^48^	40%	▲	═	▲	═	═
Zainal et al.^49^	60%	▲	═	▲	═	▲

	Rating	Groups are Randomized appropriately	Groups were comparable at baseline	Outcome data are complete	Researchers were blinded	Participants adhered to intervention
Randomized controlled trials (*n* = 1)						
Hollingworth et al.^32^	60%	▲	▲	▲	Х	═

	Rating	Adequate rationale for mixed methods	Components answer the research question	Outputs adequately interpreted	Divergences/inconsistencies addressed	Components adhere to method quality criteria
Mixed methods studies (*n* = 1)						
O'Connor et al.^60^	0%	Х	Х	Х	Х	Х

*Note*: Quality of study appraisal was conducted using the Mixed Methods Appraisal Tool (MMAT) for Qualitative, Randomized Controlled Trials and Quantitative Descriptive Studies (A–C respectively). Yes: The study described this feature within the manuscript. No: The study did not describe this feature within the manuscript. Can't tell: Raters could not agree if the authors described the feature within the manuscript. Key: ▲, Yes; Х, No; ═, can't tell.

### 3.4 Aim 1—Most and least common patient‐reported sources

#### 3.4.1 Global‐level

The 10 most reported sources of CRD, based on the NCCN PL version 2.2021,^2^ were worry (55%), fatigue (54%), fears (45%), sadness (44%), pain (41%), sleep disturbance (40%), nervousness (38%), getting around (34%), treatment decisions (30%), and dry/itchy skin (28%). The 10 least reported sources were having enough food (0%), substance abuse (3%), childbearing ability (5%), fevers (5%), spiritual problems in general (5%), housing (7%), childcare (8%), mouth sores (11%), dealing with children (11%), and sexual concerns (12%). Full results are listed in Table 4.

**TABLE 4:** Table of overall reported sources of CRD (NCCN PL version 2.2021; most to least reported).

Sources of CRD	PL domain	Articles (*n*)	Sample (*n*)	Weighted proportion (%)
Worry	Emotional	33	14,888	55
Fatigue	Physical	37	14,289	54
Fears	Emotional	37	26,121	45
Sadness	Emotional	36	26,340	44
Pain	Physical	40	16,696	41
Sleep (sleep disturbance)	Physical	42	28,341	40
Nervousness	Emotional	35	15,835	38
Getting around	Physical	30	13,864	34
Treatment decisions	Practical	12	14,389	30
Skin dry/itchy	Physical	30	13,519	28
Eating	Physical	33	15,147	27
Tingling in hands/feet	Physical	27	12,565	26
Memory/concentration	Physical	27	21,893	26
Nausea	Physical	29	13,779	24
Depression	Emotional	35	14,700	24
Constipation	Physical	31	14,984	23
Loss of interest in activities	Emotional	26	10,895	22
Indigestion	Physical	23	11,830	21
Insurance/financial	Practical	36	26,179	20
Feeling swollen	Physical	26	12,412	20
Breathing	Physical	28	13,004	18
Nose dry/congested	Physical	25	12,018	18
Changes in urination	Physical	24	12,121	17
Family health issues	Family	12	4068	16
Appearance	Physical	32	25,594	15
Diarrhea	Physical	24	11,975	14
Bathing/dressing	Physical	24	12,210	14
Dealing with partner	Family	27	13,239	13
Work/school	Practical	30	24,323	13
Transportation	Practical	28	24,039	13
Sexual	Physical	28	24,167	12
Dealing with children	Family	27	13,496	11
Mouth sores	Physical	25	12,482	11
Childcare	Practical	24	9853	8
Housing	Practical	29	24,156	7
Ability to have children	Family	9	2742	5
Fevers	Physical	23	11,935	5
Spiritual problems	Spiritual	23	10,425	5
Substance abuse	Physical	6	1672	3
Food	Practical	0	0	0

Among the eight studies using modified or alternate CRD measures, ≥25% of the pooled sample reported uncertainty (96%), coping (65%), tension (61%), hot flashes (57%), physical fitness (56%), understanding illness or treatment (39%), concentration (34%), memory (34%), emotional control (31%), making treatment decisions (30%), muscle strength (30%), householding (28%), and sore/dry mouth (27%) as sources of CRD (Table 5).

**TABLE 5:** References of included studies, listed by continent.

Continent	Country	Reference
Africa	Cameroon	Ebob‐Anya et al.^28^
Africa	Egypt	Abd El‐Aziz et al.^23^
Asia	China	Guan et al.^30^
Asia	China	Hong et al.^57^
Asia	China	Hong et al.^33^
Asia	China	Tan et al.^67^
Asia	Iran	Saedi et al.^44^
Asia	Korea	Park et al.^64^
Asia	Malaysia	Zainal et al.^49^
Asia	Singapore	Mahendran et al.^36^
Asia	Vietnam	Nguyen et al.^41^
Europe	Croatia	Budisavljevic et al.^53^
Europe	Denmark	Mertz et al.^55^
Europe	Germany	Goebel et al.^50^
Europe	Germany	Mehnert et al.^39^
Europe	Germany	Mehnert et al.^68^
Europe	Germany	Peters et al.^43^
Europe	Greece	Kyranou et al.^35^
Europe	Italy	Bellè et al.^25^
Europe	Norway	Seland et al.^61^
Europe	The Netherlands	Braamse et al.^63^
Europe	The Netherlands	Jacobs et al.^58^
Europe	The Netherlands	Roerink et al.^16^
Europe	UK	Hollingworth et al.^32^
Europe	UK	Rooney et al.^52^
N. America	Canada	Blais et al.^27^
N. America	Canada	Giese‐Davis et al.^29^
N. America	Canada	Naik et al.^56^
N. America	Mexico	Ascencio‐Huertas et al.^24^
N. America	United States	Graves et al.^66^
N. America	United States	Hegel et al.^54^
N. America	United States	Hildenbrand et al.^31^
N. America	United States	Jewett et al.^59^
N. America	United States	McDonagh et al.^38^
N. America	United States	Randazzo et al.^51^
N. America	United States	Tonsing and Vungkhanching^45^
N. America	United States	Troy et al.^69^
N. America	United States	Troy et al.^65^
N. America	United States	VanHoose et al.^46^
N. America	United States	Wookey and McKean^48^
Oceania	Australia	Kirk et al.^34^
Oceania	Australia	Musiello et al.^40^
Oceania	Australia	O'Connor et al.^60^
Oceania	Australia	Opie et al.^42^
Oceania	Australia	Watts et al.^47^
S. America	Brazil	Bergerot et al.^26^
S. America	Brazil	Bergerot et al.^62^
S. America	Brazil	Mendonça et al.^37^

*Note*: Tables showing comprehensive results for continent‐level analyses are available from the first author by request.

#### 3.4.2 Continent‐level

For North American studies (*n* = 15), the five most reported sources of CRD were fatigue (62%), worry (58%), sadness (48%), fears (47%), and nervousness (46%). Among European studies (*n* = 14), fatigue (52%), worry (48%), sleep disturbance (46%), fears (41%), and getting around (40%) were the five most reported sources of CRD. The five most reported sources of CRD in Asian studies (*n* = 9) were worry (55%), fatigue (45%), pain (45%), sleep disturbance (41%), and depression (41%). Among studies conducted in Oceania (*n* = 5), the five most reported sources of CRD were worry (73%), fatigue (64%), fears (50%), sadness (50%), and nausea (50%). Among studies conducted in South America (*n* = 3), the five most reported sources of CRD were worry (68%), nervousness (56%), sadness (50%), sleep disturbance (47%), and fears (46%). In African studies (*n* = 2), the five most reported sources of CRD were treatment decisions (64%), fatigue (53%), worry (48%), fears (44%), and pain (43%).

### 3.5 Aim 2—Sources of CRD and actionable CRD

Fifteen studies observed relationships between individual reported sources of CRD and reporting actionable levels of CRD severity. Emotional concerns of sadness,^23^, ^24^, ^34^, ^35^, ^36^, ^37^, ^42^, ^45^, ^53^, ^61^, ^64^, ^68^ nervousness,^24^, ^35^, ^36^, ^41^, ^42^, ^44^, ^45^, ^53^, ^61^, ^64^, ^68^ depression,^23^, ^34^, ^35^, ^37^, ^41^, ^42^, ^44^, ^53^, ^61^, ^64^ fears,^23^, ^35^, ^37^, ^42^, ^44^, ^45^, ^53^, ^61^, ^64^, ^68^ worry,^23^, ^34^, ^35^, ^37^, ^42^, ^45^, ^53^, ^61^, ^64^, ^68^ and loss of interest in usual activities^23^, ^34^, ^35^, ^42^, ^53^, ^61^, ^64^ were found to be positively associated with actionable CRD severity across at least four studies. Among physical concerns, pain,^35^, ^41^, ^42^, ^44^, ^45^, ^53^, ^58^, ^61^, ^64^, ^68^ sleep disturbance,^23^, ^35^, ^41^, ^42^, ^44^, ^45^, ^53^, ^61^, ^64^, ^68^ fatigue,^35^, ^41^, ^42^, ^44^, ^45^, ^53^, ^58^, ^68^ eating,^24^, ^35^, ^37^, ^41^, ^44^, ^53^, ^64^ memory/concentration,^41^, ^42^, ^44^, ^53^, ^61^ appearance,^35^, ^42^, ^44^, ^53^ changes in urination,^36^, ^37^, ^44^, ^68^ constipation,^35^, ^36^, ^42^, ^64^ were found to be positively associated with actionable CRD severity in four or more studies. Among practical concerns, at least four studies found housing,^24^, ^35^, ^41^, ^42^, ^53^, ^64^ insurance/financial,^23^, ^41^, ^42^, ^53^ and work/school^41^, ^42^, ^53^, ^68^ to be positively associated with actionable CRD severity. No family or spiritual concerns were reported by four or more studies. All significant relationships reported in each study are listed in Table 1.

### 3.6 Aim 3—Sources of CRD and demographic and clinical characteristics

#### 3.6.1 Sources of CRD and demographics

Emotional concerns were related to age, sex, marital status, income, education, and rurality in 10 studies. In general, emotional concerns were negatively correlated with age,^36^, ^56^ income,^67^ and education.^36^, ^67^ Women were found to report concerns from emotional,^27^, ^39^, ^43^, ^51^, ^62^ and psychosocial^29^ domains more frequently than men. Among specific emotional concerns, worry and fears were more frequently reported by people with lower incomes,^59^ people living in rural areas,^47^ and people with lower/informal education.^36^, ^67^ Nervousness was more frequently reported by partnered individuals,^59^ people with lower incomes,^59^ and people with lower/informal education.^36^

Physical concerns were related to age, sex, race/ethnicity, income, and rurality in nine studies. Reporting fatigue as a source of CRD was negatively related to age and income.^59^ Women reported physical concerns^27^, ^43^, ^51^, ^62^ more frequently than men with exception of changes in urination,^39^ and sexual concerns.^29^, ^39^, ^51^ Physical concerns were more frequently reported among the ethnic majority in Malaysia^36^ and among African Americans (specifically, changes in urination, memory/concentration, fertility, and sexual problems).^38^ Physical concerns about pain, mouth sores, and nausea were more frequently reported by people living in rural areas.^47^

Practical concerns were related to age, sex, race/ethnicity, marital status, rurality, and education in seven studies. Practical concerns were more frequently reported by young adults,^56^ men,^43^, ^51^ African Americans,^38^ and single individuals.^29^ Among specific concerns, insurance and transportation were more frequently reported by African Americans^38^ and people living in rural areas.^47^ Concerns about work/school more frequently reported by African Americans.^38^ Concerns about childcare were more frequently reported by people with secondary education.^36^

Family concerns were related to age and sex in five studies, with more frequent reports by young adults^56^ and women.^27^, ^36^, ^39^, ^51^ Spiritual concerns were related to sex and race/ethnicity in two studies,^27^, ^38^ with spiritual concerns being reported more frequently by women^56^ and African Americans.^38^

#### 3.6.2 Sources of CRD and clinical characteristics

Emotional concerns were related to treatment type and insurance in three studies. Emotional concerns were reported more frequently by people receiving chemotherapy,^59^ especially when not preceded by surgery or radiation therapy.^36^ Specific concerns of sadness, fears, and nervousness were reported more frequently by people receiving chemotherapy,^59^ while worry was reported less frequently by people who had surgery and those with private health insurance versus government‐provided insurance.^36^, ^67^

Physical concerns were related to treatment type, cancer grade, and time of screening in four studies. Pain and fatigue were more likely to be reported by people receiving chemotherapy,^59^ while physical concerns were reported less frequently by people who had surgery.^36^ Physical concerns were reported more frequently by people with high‐grade cancers^51^, ^62^ and those who received CRD screening prior to seeing the oncologist.^36^

Practical concerns were related to treatment type, cancer grade, and performance status in four studies. Practical concerns were more frequently reported by people with high‐grade cancers^51^, ^62^ and poorer performance status.^50^ Concerns about childcare were more frequently reported by people receiving treatments other than chemotherapy.^53^ No significant relationships were reported between family or spiritual concerns and clinical characteristics across studies.

## 4 DISCUSSION

### 4.1 Most/least frequent sources of CRD

Findings demonstrate common reported sources of CRD with relative consistency across continents. More than 40% of people across studies reported worry, fatigue, fears, sadness, pain, and sleep disturbance as sources of CRD. Clinicians can anticipate that people with cancer are likely to report these common sources as contributing to their CRD and prepare available interventions in advance. Cancer care organizations should consider allocating resources toward implementing interventions known to be beneficial for management of common sources of CRD, such as physical activity, guided imagery or mindfulness for management of CRD from fatigue and sleep disturbance.^70^ Researchers should continue to develop and test interventions targeting common sources of CRD, emphasizing interventions that can be delivered at the time of screening without increased burden to healthcare providers.^71^, ^72^, ^73^ Providing interventions at the time of screening may reduce reliance on referrals to social workers, health psychologists, and/or other specialists, which people with cancer frequently decline or fail to follow‐up.^12^, ^13^, ^74^

Twenty‐seven of the forty sources of CRD on the PL (version 1.2021) were reported by less than 25% of pooled samples, with ≤5% reporting concern about lack of food, substance use, spiritual problems, fevers, and childbearing ability. Depression, often used as a proxy measure for CRD, was reported by less than 25% of the pooled sample and only appeared among the top 10 reported sources of CRD in studies conducted in Asian countries. Low reporting of depression suggests that organizations using depression measures, such as the Patient Health Questionnaire,^75^ to screen for CRD risk failing to identify a majority of people with CRD from other sources. This is consistent with research demonstrating that the NCCN DT/PL was able to detect CRD missed by measures of depression or anxiety^14^ and strengthens guidance recommending use of CRD‐specific screening tools.^71^

Our review invites consideration to refine the sources of CRD that are included in standard screening measures. The NCCN PL recently underwent major revision, eliminating many physical concerns, adding sources of CRD to practical and spiritual concerns, modifying the family domain to a more inclusive social domain, and relocating some sources of CRD to different domains.^20^ While the most highly reported physical concerns of fatigue, pain, and sleep disturbance remain, a number of the retained physical concerns had low frequency of reporting in the current review, for example, substance abuse (3%) and sexual concerns (12%). Conversely, three physical concerns removed from the NCCN PL were reported by at least 25% of the pooled sample in the current review, for example, getting around (34%), dry/itchy skin (30%), tingling in hands/feet (26%). Having enough food, though not reported as a source of CRD across included studies, was retained on the updated PL. Finally, six spiritual concerns were added, addressing aspects of spirituality broadly from dietary considerations to concern about death and dying. In our review, spiritual concerns (as a single item) were reported by only 5% of pooled samples. The addition of new sources of CRD in this domain may reveal higher endorsement in future studies. Some CRD sources such as access to food, childcare, substance use, and financial toxicity merit inclusion for even low levels of endorsement to facilitate treatment adherence and mitigate potential harms.^76^, ^77^ PLs using a set of common core sources of CRD augmented with CRD sources of significant consequence or important for select patient subgroups (e.g., diagnosis or therapy‐specific) may enhance usefulness in high‐risk groups while minimizing screening burden for others.

### 4.2 CRD sources related to actionable levels of CRD severity

All emotional concerns and select concerns in physical, practical, and family domains were positively related to actionable CRD severity. Based on observed findings, clinicians could make reasonable inference that reporting any emotional concern could indicate the presence of actionable CRD severity. Similarly, people experiencing pain, sleep disturbance, fatigue, eating difficulties, problems with memory or concentration, housing issues, financial concerns, and/or domestic partner concerns could be considered at increased risk for actionable CRD. Moving concerns with low association with actionable CRD, such as having enough food and substance use, to a supplemental module could be considered, particularly if the intent is to assess for the presence of the condition (identifying unmet need), rather than distress from it. While CRD severity is known to be positively correlated with greater numbers of unmet needs, specific needs contributing to CRD (sources of CRD) vary by clinical and demographic characteristics.^78^, ^79^, ^80^ For example, a person may use substances, such as use of cannabinoids for management of appetite, nausea, or pain, but not be distressed by it.

### 4.3 CRD sources related to clinical and demographic characteristics

Differences in reported sources of CRD and demographics, specifically age, sex, marital status, income, rurality, and education were identified. In general, being male, older, and partnered reduced frequency of reporting emotional, physical, family, and practical concerns. Younger people and people with lower education and/or income appeared to report more emotional and practical concerns. Although the majority of study samples were White and urban‐dwelling, our findings suggest that race/ethnicity and rurality may contribute to differences in reported sources of CRD.

Relationships were also identified between reported sources of CRD and clinical characteristics including cancer grade, treatment type, insurance, performance status, and timing of screening. Higher grade cancers were associated with more physical and practical concerns. Receiving chemotherapy was associated with more emotional and physical concerns, but fewer practical concerns (childcare) than other treatment types. Having private health insurance was associated with lower endorsement of worry as a source of CRD. Poor performance status was associated with greater endorsement of practical concerns. Physical concerns were more frequently reported by people who were screened for CRD before seeing their oncologist.

While our synthesis summarized evidence regarding bivariate relationships, it is likely that relationships among sources of CRD and demographic and clinical characteristics are far more complex. For example, Giese‐Davis and colleagues^29^ observed age and gender to interact in their relationship with sources of CRD, finding that younger females were significantly more likely to report psychosocial problems than older males; the number of psychosocial concerns reported decreased over time as age increased; and males report significantly fewer concerns than females as age increased. Demographic and clinical characteristics may be related to these patterns of reported sources of CRD.

People with cancer have reported multiple co‐occurring, interrelated sources of CRD. Physical and emotional effects of cancer and treatment (inflammation, activated hypothalamic procedures, etc.) are thought to contribute to clustering sources contributing to CRD, such as pain, depression, and fatigue.^81^, ^82^ Jacobs and colleagues identified CRD problem clusters, including fears and tension, weight changes and eating, and physical fitness and fatigue.^58^ CRD symptom clusters were identified among head & neck cancer survivors relating to adjustment (appearance, breathing, and worry), rurality (speech, getting around, and family illness), and others.^83^ Many of the most reported sources of CRD are also found within common symptom clusters, such as co‐occurring pain, fatigue, and sleep disturbance.^84^, ^85^ Further research is needed to evaluate the contribution of symptom clusters as sources of CRD and the effect of CRD on the experience of multiple co‐occurring symptoms.

A more sophisticated analysis, which takes complexities of relationships into account, such as latent class modeling, may be helpful for identifying common characteristics of people who report similar patterns in sources of CRD. For example, a young Asian woman with breast cancer will likely have a different CRD profile than an older African American male with prostate cancer.^86^, ^87^ Current practice relies on risk stratification for intervention based on CRD severity using the CRD severity exceeding a cut‐off score (usually DT ≥4),^1^ with people reporting sub‐clinical CRD (severity of three or less) may not receive any follow‐up, regardless of the sources of CRD reported. Our findings indicate that some sources of CRD may be more distressing than others and may be appropriate for intervention regardless of reported CRD severity. Research has demonstrated that lack of appropriate aftercare is the most significant barrier to CRD screening program implementation and screening with immediate intervention/resources is more effective than screening alone.^88^, ^89^ Greater understanding of common sources of CRD and, and possibly CRD profiles, could facilitate an improved risk‐stratified approach to CRD screening and management by allowing to be proactive in offering targeted psychosocial interventions for the common sources of CRD. Greater understanding of sources of CRD can enhance research in CRD management by clarifying targets for intervention development.

### 4.4 Strengths and limitations

This review has a number of strengths. First, this review represents a novel line of inquiry. To our knowledge, no other review has synthesized the available evidence about common patient‐reported sources of CRD, associations between sources of CRD and actionable CRD, or associations between sources of CRD and demographic or clinical characteristics. This analysis is critical for understanding the overall picture of patient‐reported sources of CRD not attainable from single studies or quality improvement projects. Second, we synthesized evidence from literature on a global scale. The majority of studies reporting sources of CRD (*n* = 37) were conducted outside the United States. We present results at the continent‐level to assist researchers and clinicians in transferring the knowledge to their respective contexts. Third, weighting the data by sample size during analysis reduced risk for data from smaller studies to overexert influence on the results. Finally, we employed a rigorous methodology for screening, data extraction, data analysis, and quality assessment.

Findings of this review should be interpreted in context of some limitations. It is possible that our review failed to capture every study that reported common sources of CRD. Further, we limited our review to studies reported in English; thus, our findings reported by continent may exclude relevant studies published in other languages. Several studies had to be excluded due to incomplete reporting of sources of CRD which may result in over‐or under‐representation in our synthesis. Several studies reported only statistically significant associations between demographic and/or clinical characteristics, potentially underrepresenting null findings.

In conclusion, this review found commonly reported sources of CRD, specifically worry, fatigue, sleep disturbance, fears, and sadness, that were generally consistent across global locations. In the clinical setting, healthcare providers can anticipate that many of the people in their care will experiencing CRD from one or more of these sources. Resources should be allocated toward implementation of interventions for these concerns which can be administered by providers at the time of screening and point of care. Moreover, results can be used to refine screening tools, informing a core set of CRD sources for all persons with cancer and supplemental items for specific diagnoses, treatment approaches, or risk categories. Finally, results of this review can inform future research to identify people at risk for CRD by examining shared patterns in reported sources of CRD and associated demographic and clinical characteristics and directing development of targeted interventions.

## AUTHOR CONTRIBUTIONS

**Jennifer M. Stevens:** Conceptualization (lead); data curation (lead); formal analysis (lead); funding acquisition (equal); investigation (equal); methodology (equal); project administration (lead); resources (equal); software (lead); supervision (equal); validation (equal); visualization (equal); writing – original draft (lead); writing – review and editing (lead). **Kathleen Montgomery:** Conceptualization (supporting); methodology (supporting); validation (equal); visualization (equal); writing – review and editing (supporting). **Megan Miller:** Conceptualization (supporting); validation (equal); writing – review and editing (supporting). **Seyedehtanaz Saeidzadeh:** Formal analysis (supporting); validation (equal); visualization (supporting); writing – review and editing (supporting). **Kristine L. Kwekkeboom:** Conceptualization (equal); formal analysis (supporting); funding acquisition (equal); investigation (supporting); methodology (equal); project administration (equal); resources (supporting); supervision (equal); validation (equal); visualization (equal); writing – original draft (supporting); writing – review and editing (equal).

## FUNDING INFORMATION

Jennifer Stevens is supported by The American Cancer Society – Coaches vs, Cancer Bo Ryan Jay Holliday Families Fund Doctoral Degree Scholarship (DSCN‐20‐093‐01).

## CONFLICT OF INTEREST STATEMENT

No authors have known conflicts of interests to disclose.

## PRECIS

This systematic review identifies the most and least frequently reported sources of cancer‐related distress. Differences reported in the number and type of sources of cancer‐related distress according to demographic and clinical characteristics are synthesized.

## Data Availability

Data available on request from the authors.
